# Vaccination of pregnant women: an overview of European policies and strategies to promote it

**DOI:** 10.3389/fpubh.2024.1455318

**Published:** 2024-12-09

**Authors:** S. Properzi, R. Carestia, V. Birettoni, V. Calesso, B. Marinelli, E. Scapicchi, E. Brillo, C. de Waure

**Affiliations:** ^1^Department of Medicine and Surgery, University of Perugia, Perugia, Italy; ^2^Department of Medicine and Surgery, School of Midwifery, University of Perugia, Perugia, Italy; ^3^Center for Research in Perinatal and Reproductive Medicine, University of Perugia, Perugia, Italy

**Keywords:** vaccination, vaccine policies, knowledge, behavior, pregnancy

## Abstract

Maternal immunization is a valuable tool for protecting mother and unborn child from vaccine-preventable diseases. However, the implementation of strategies for vaccinating pregnant women has only recently gained traction. This work is aimed at providing an overview of European vaccination strategies and gathering evidence on interventions enhancing vaccination knowledge, attitudes, and behaviors (KAB) in pregnant women. To summarize current pregnancy vaccination strategies in Europe, we consulted literature, institutional national health system websites, and the ECDC Vaccine Scheduler. The review of evidence on interventions targeting pregnant women’s vaccination KAB was performed by searching primary studies on PubMed and Web of Science. The 27 EU member states offer various vaccinations in pregnancy, but only 10 recommend all of these: tetanus, pertussis, diphtheria, influenza, and COVID-19, albeit with different administration schedules. The literature review included 7 studies, 3 from Italy and 4 from other European countries (UK, Netherlands, Greece, Poland, and Ukraine). They were conducted in various settings such as childbirth preparation courses, prenatal visits, and online platforms, and all included educational interventions providing information on vaccine safety and efficacy during pregnancy. Knowledge about vaccines and vaccine-preventable diseases, generally low in the pre-intervention period, increased post-intervention, with a rise in awareness of the risks associated with infectious diseases and the recommended vaccines, a reduction in vaccine-related misinformation, and a greater propensity to vaccinate both newborns and themselves. Furthermore, there was a significant increase in adherence to recommended vaccinations, particularly among those with higher educational levels. However, vaccine hesitancy persisted, influenced by factors such as fear of adverse events and the lack of recommendations from healthcare providers. Variations in pregnancy vaccination strategies across Europe emphasize the importance of establishing a unified framework to optimize maternal and fetal health outcomes through evidence-based policies. Educational interventions may positively impact pregnant women’s KAB, therefore promoting vaccination uptake.

## 1 Introduction

Throughout pregnancy, the immune system undergoes significant modulation alongside physiological adaptations aimed at maintaining maternal homeostasis and facilitating optimal fetal development. These alterations make women more vulnerable to both viral and bacterial infections (1–3), consequently heightening the likelihood of severe complications for the mother and the potential transmission of pathogens to the developing fetus (4–6).

Due to the immaturity of their immune system in the first months of life, neonates are notably susceptible to the onset of potentially severe or fatal infections until they reach the age suitable for vaccination and complete the vaccination cycle (7).

Vaccinating pregnant women has been identified as an optimal strategy for safeguarding the health of the mother, fetus, and infant, resulting in a triple benefit. This intervention affords pregnant women, protection against vaccine-preventable diseases (VPDs) such as influenza, diphtheria, tetanus, pertussis, and COVID-19 (8, 9). Furthermore, a vaccine against Respiratory Syncytial Virus (RSV) has been recently approved in pregnant women for the protection of infants from lower respiratory tract diseases (10).

Therefore, vaccination in pregnancy is widely recognized as an essential component of the comprehensive antenatal care package aimed at enhancing maternal and child health (11, 12).

In this light, many European countries followed the guidance provided by the World Health Organization (WHO) (13–15) and the Advisory Committee on Immunization Practices (ACIP) of the Centers for Disease Control and Prevention (CDC) (16, 17), routinely advocate for maternal immunization to prevent influenza, diphtheria, pertussis, tetanus, and COVID-19, often through fully subsidized vaccine offerings, as evidenced by a comprehensive review of vaccination policies specific to pregnant women in Europe published in 2021 (18). These vaccines have been demonstrated safe, immunogenic, and effective (19). Nevertheless, vaccine coverage in Europe among pregnant women exhibits substantial discrepancies in terms of both monitoring and data (20). The 2018 ECDC report indicated that only nine European Union Member States (21), reduced to four in the most recent 2023 report (22), monitored pregnant women’s adherence to seasonal influenza vaccination. The highest influenza vaccination rates were observed in Northern Ireland (58.6%) and England (44.9%) during the 2016–2017 influenza season, while Ireland reached 62% in 2017–2018 (21). A wide variability in influenza vaccination coverage, ranging from 1.7 to 61%, was indeed shown in 2020–2021 (22). Significant variability was evident also in respect to other vaccinations, such as pertussis, with high vaccination coverage in Spain, Denmark, and Belgium (88.5, 69, and 64.3%, respectively), in stark contrast to the low ones observed in the Czech Republic and Slovenia (1.6 and 6.5%) in 2023 (23).

Regarding SARS-CoV-2 during the 2023–2024 season, only Ireland (19.6%) and Spain (7.8%) have published official data (24), emphasizing the considerable efforts still required, not only to achieve adequate vaccination coverage in this at-risk population but also to ensure effective monitoring.

The substantial variability in vaccination coverages and their unsatisfactory level can be partly attributed to “vaccine hesitancy” (25), which is defined by the WHO’s Strategic Advisory Group of Experts on Immunization (SAGE) (26) as the inclination to postpone or decline vaccination despite its availability and is currently recognized as one of the top ten threats to global health (27, 28).

Several studies have explored the factors that influence vaccine hesitancy in pregnancy. These investigations have consistently identified some elements in the literature, namely vaccine-specific factors, such as fear of adverse events and lack of confidence in vaccine safety, and lack of recommendation from healthcare professionals. Disease-related perceptions as well as previous vaccination behavior have also been shown to have an impact on vaccine uptake (9, 29, 30).

This evidence underscores the imperative need to address the determinants influencing maternal immunization, including knowledge, attitudes, and beliefs about maternal and childhood vaccines, through educational interventions (19, 31–34). Such measures are crucial to promoting behavioral changes in pregnant women and their families, enhancing adherence to vaccination protocols, and thus reducing vaccine hesitancy in pregnancy (35, 36).

This review aims to provide an updated overview of pregnant women’s vaccination policies across Europe and of current evidence regarding educational interventions aimed at promoting knowledge, attitudes, and behaviors related to recommended vaccinations for pregnant women in the European context. Based on the identified issues and problems the paper seeks to explore potential avenues for optimizing maternal and fetal health outcomes within diverse European settings.

## 2 Materials and methods

To procure a contemporaneous assessment of extant vaccination strategies tailored for pregnant women in Europe, we consulted the “Vaccine Scheduler” of the ECDC (37). Additionally, we examined the recommendations provided by national health systems, as available on their institutional websites, or reported in the comprehensive review of pregnancy vaccination policies in Europe published in 2021 (18).

Moreover, a review focusing on educational interventions aimed at promoting knowledge, attitudes, and behaviors regarding recommended vaccinations among pregnant women, namely influenza, diphtheria, tetanus, pertussis, and COVID-19, was conducted. Educational interventions have been considered in various formats, including, for example, expert-led information sessions, digital campaigns, and distribution of themed information materials. The primary objective of the search was to identify studies that assessed the impact of these interventions on pregnant women’s knowledge, attitudes, and behaviors toward vaccination recommended in pregnancy. To achieve this objective, we employed a search string and adhered to the PICOS criteria, although we did not intend to conduct a systematic review. The evidence retrieval was conducted by consulting two databases (MEDLINE/PubMed, and Web of Science) up to 21 May 2023. Search terms related to pregnancy, vaccination, immunization, knowledge, attitudes, and behaviors regarding vaccination were included. Only language filters were applied to include articles in English, French, and Italian.

The entire search strategy is reported in Table 1.

**Table 1:** Search strategy.

Search engine	Search strategy
PubMed	(strategy[Title/Abstract] OR intervention[Title/Abstract] OR program[Title/Abstract]) AND (vaccination[Title/Abstract] OR immunization[Title/Abstract]) AND (pregnancy[Title/Abstract] OR pregnant[Title/Abstract] OR antenatal[Title/Abstract] OR ante-partum[Title/Abstract]) AND (knowledge[Title/Abstract] OR attitudes[Title/Abstract] OR behaviour[Title/Abstract] OR belief[Title/Abstract] OR coverage[Title/Abstract] OR uptake[Title/Abstract] OR trust[Title/Abstract] OR mistrust[Title/Abstract] OR perception[Title/Abstract] OR hesitancy[Title/Abstract] OR confidence[Title/Abstract] OR acceptance[Title/Abstract] OR adherence)[Title/Abstract]
WoS	(TS = (strategy OR intervention OR program)) AND (TS = (vaccination OR immunization)) AND (TS = (pregnancy OR pregnant OR antenatal OR ante-partum)) AND (TS = (knowledge OR attitudes OR behaviour OR belief OR coverage OR uptake OR trust OR mistrust OR perception OR hesitancy OR confidence OR acceptance OR adherence))

The inclusion criteria for studies were based on the PICOS framework (38), as described below: (P) Population: European pregnant women during any trimester of pregnancy; (I) Intervention: any intervention involving education, training, or vaccination awareness initiatives; (C) Comparison: not applicable; (O) Outcome: knowledge, attitudes, and behaviors of women toward vaccinations; (S) Study design: primary studies with experimental or quasi-experimental designs, including randomized and non-randomized trials, and observational studies.

The PICOPortal platform (39) was used for screening and for identifying duplicates. Records underwent initial screening by two reviewers, with a third reviewer resolving equivocal cases. The full texts of selected articles were independently reviewed by two reviewers for eligibility.

Within the scope of this narrative review, a qualitative synthesis was conducted. Information about the study setting, the study population, the sample size, the type of intervention, and the tools used to assess the impact of the intervention were extracted by each study by a researcher and cross-checked by a second one. Data about pregnant women’s knowledge, attitudes, and behaviors were also collected from each study and reported descriptively highlighting any significant difference due to the intervention. We employed the NIH quality assessment tools, specifically the “Quality Assessment Tool for Before-After (Pre-Post) Studies With No Control Group” and the “Quality Assessment of Controlled Intervention Studies” to evaluate the quality of the included studies (40). The former tool evaluates pre-post studies by examining 12 aspects such as the clarity of study objectives, the inclusion of pre-specified outcome measures, the appropriateness of statistical analysis, and the consideration of potential confounding factors. Three distinct categories were identified based on the scoring: 0–4 as poor, 5–8 as fair, and 9–12 as good. The second tool assesses controlled intervention studies based on 14 key criteria such as randomization, allocation concealment, blinding, completeness of outcome data, selective reporting, and other sources of bias. Also in this case, three quality categories were identified based on the scoring: 0–4 as poor, 5–9 as fair, and 10–14 as good.

## 3 Results

### 3.1 Overview of vaccination policies in Europe

Despite the diversity of vaccination programs, several European countries implement tailored vaccination policies for pregnant women (18), following guidelines outlined by the WHO (13–15). Nevertheless, strategies exhibit variability across European Countries (17, 32).

An examination of the most recent directives from 39 states, including European Union member states, revealed that 97% (38) of such states advocate for the administration of the influenza vaccine during the gestational period. Furthermore, 77% (30) endorse vaccination against pertussis, with 38% (15) advocating for the tetanus vaccine, 28% (11) for the diphtheria vaccine, and 56% (22) for vaccination against COVID-19. Lastly, 26% (10) endorse the entirety of the aforementioned vaccinations for women in a pregnant state (Table 2) (18, 37).

**Table 2:** Vaccination programs for pregnant women in Europe.

Country	Influenza	Pertussis	Coronavirus	Tetanus	Diphtheria
Belgium^EU^	2nd–3rd trimester	24th–32nd week	1st–3rd trimester	24th–32nd week	24th–32nd week
Spain^EU^	1st–3rd trimester	From 27th week	1st–3rd trimester	From 27th week	From 27th week
Bulgaria^EU^	2nd–3rd trimester	27th–36th week		2nd–3rd trimester	2nd–3rd trimester
Ireland^EU^	1st–3rd trimester	2nd–3rd trimester	1st–3rd trimester	2nd–3rd trimester	2nd–3rd trimester
Italy^EU^	1st–3rd trimester	3rd trimester	1st–3rd trimester	3rd trimester	3rd trimester
Finland^EU^	1st–3rd trimester	From 16th to 32nd week	1st–3rd trimester		
Estonia^EU^	1st–3rd trimester	2nd–3rd trimester			
Croatia^EU^	1st–3rd trimester	2nd–3rd trimester			2nd–3rd trimester
Germany^EU^	2nd–3rd trimester*	2nd–3rd trimester	2nd trimester		3rd trimester**
Norway	2nd–3rd trimester*	From 24th week	2nd–3rd trimester	2nd–3rd trimester	2nd–3rd trimester
Denmark^EU^	2nd–3rd trimester*	24th–32nd week**	1st–3rd trimester		
Netherlands^EU^	2nd–3rd trimester	From 22nd week		From 22nd week	From 22nd week
Luxemburg^EU^	1st–3rd trimester	13th–26th week	From 10th week		
Portugal^EU^	2nd–3rd trimester	20th–36th week	1st––3rd trimester		
Iceland	1st–3rd trimester	2nd–3rd trimester	1st–3rd trimester		
Switzerland	1st–3rd trimester	13th–26th week	From 13th week		
Sweden^EU^	2nd–3rd trimester	From 16th week	From 12th week		
Austria^EU^	2nd–3rd trimester*	27th–36th week	2nd–3rd trimester		
Czech Republic^EU^	1st–3rd trimester	3rd trimester	From 13th week		
France^EU^	1st–3rd trimester	2nd–3rd trimester	1st–3rd trimester		
Romania^EU^	1st–3rd trimester	2nd–3rd trimester			
Ukraine	1st–3rd trimester	3rd trimester			
Cyprus^EU^	1st–3rd trimester	27th–36th week			
Greece^EU^	1st–3rd trimester	27th–36th week			
Poland^EU^	1st–3rd trimester	27th–36th week			
Liechtenstein	1st–3rd trimester	2nd trimester			
Slovenia^EU^	1st–3rd trimester	From 24th week			
United Kingdom	1st–3rd trimester	2nd–3rd trimester			
Serbia	1st–3rd trimester	3rd trimester			
Lithuania^EU^	1st–3rd trimester		1st–3rd trimester		
Slovakia^EU^	1st–3rd trimester		1st–3rd trimester		
Malta^EU^	2nd–3rd trimester*		From 12th week		
Moldova		3rd trimester			
Albania	1st–3rd trimester				
Belarus	1st–3rd trimester				
Hungary^EU^	1st–3rd trimester				
Latvia^EU^	1st–3rd trimester				
Monaco	1st–3rd trimester				
Russia	2nd–3rd trimester*				

Dark grey: Recommended for all pregnant women. Light grey: Recommended in specific situations: epidemics or at-risk conditions. *extended to 1^st^ trimester only in women with high-risk conditions ** extended to 2nd trimester only in women with increased risk of premature birth.

Thirty-eight European countries advocate for administering the influenza vaccine to pregnant women, though with different timings (18, 37). Notably, Belgium, Bulgaria, the Netherlands, Portugal, and Sweden recommend influenza vaccine in the 2nd–3rd trimester. Austria, Denmark, Germany, Malta, Norway, and Russia also stipulate that influenza vaccination is advisable for pregnant women in the 2nd to the 3rd trimester (18, 41–45), but extend their recommendation to include vaccination from the onset of the 1st trimester in pregnant women with high-risk conditions or during epidemics (18, 37). Twenty-seven out of the 38 countries (Albania, Belarus, Croatia, Cyprus, Czech Republic, Estonia, Finland, France, Greece, Hungary, Iceland, Ireland, Italy, Latvia, Liechtenstein, Lithuania, Luxemburg, Monaco, Poland, Romania, Serbia, Slovakia, Slovenia, Spain, Switzerland, Ukraine, and the United Kingdom), recommend influenza vaccination between the 1st and 3rd trimester (18, 37).

Pertussis vaccination is also advised during pregnancy in numerous European countries, with notable variations in the timing and condition of recommendation. Luxembourg and Switzerland recommend vaccination between the 13th and 26th weeks, Sweden and Finland from the 16th week, Portugal between the 20th and 36th week, Denmark and Belgium between the 24th and 32nd week, the Netherlands from the 22nd week, Slovenia and Norway from the 24th week and Austria, Bulgaria, Cyprus, Czech Republic, Germany, Greece, Italy, Poland, Serbia, Spain and Ukraine from the 27th week (18, 37, 46–54). In Denmark, as well as in Germany, vaccination is extended at the beginning of the 2nd trimester if premature labor is expected (18, 37, 52). Estonia, Iceland, Ireland, and the United Kingdom recommend vaccination between the 2nd and 3rd trimester, as well as Romania if more than 10 years have elapsed after the last dose (18, 37, 55, 56). In Liechtenstein, pertussis vaccination is advocated during the 2nd trimester (18). Few countries recommend the vaccination in response to prevailing epidemiological trends, such as Moldova (recommended in the 3rd trimester during epidemics or high-risk conditions), France (recommended in the 2nd–3rd trimester in the epidemic territory), Croatia (recommended in the 2nd–3rd trimester in light of the ongoing pertussis epidemic) (37).

As far as diphtheria vaccination is concerned, in Bulgaria and Ireland it is recommended between the 2nd and the 3rd trimester of pregnancy, along with tetanus vaccination (18, 37). In the Netherlands, the diphtheria vaccination is advised from the 22nd week of pregnancy, in Belgium between the 24th and 32nd week, in Spain and Italy in the 3rd trimester, ideally from the 27th week and at the 28th week, respectively (18, 37). In these countries, tetanus vaccination is also recommended in the same time window (18, 37, 57–61). In Finland, vaccination against diphtheria is recommended for all pregnant women, preferably at the end of pregnancy (18). In Germany, vaccination against diphtheria is advocated at the beginning of the 3rd trimester, and extended at 2nd in women at risk of pre-term birth (41), while in Estonia it is recommended for women presenting specific risk conditions (18); furthermore, in these countries, as well as in Finland, Denmark, Moldova, Romania, and Ukraine, tetanus vaccination is recommended for pregnant women who are either unvaccinated or incompletely vaccinated, as well as for pregnant women following exposure to potential tetanus risks (18). In Norway, the consideration for administering the diphtheria vaccine arises if clinically warranted; it is prudent to defer vaccination until the 2nd–3rd trimester rather than administering it during the initial trimester (18). Additionally, Norway recommends tetanus vaccination between the 2nd and the 3rd trimesters, specifically during epidemics or for individuals with risk conditions (18).

Croatia temporarily advises diphtheria and tetanus vaccination for all pregnant women during the 2nd–3rd trimester, along with vaccination for all close contacts of newborns (37).

COVID-19 vaccination is recommended for pregnant women across all trimesters in 14 European countries (Belgium, Bulgaria, Croatia, Denmark, Estonia, Finland, France, Iceland, Ireland, Italy, Lithuania, Portugal, Slovakia, Spain) (18, 37). On the contrary, in Luxembourg, it is suggested starting from the 10th week of pregnancy (62), while in Malta and Sweden, the recommendation begins from the 12th week (63, 64). In the Czech Republic, COVID-19 vaccination during pregnancy is deemed particularly appropriate for women exhibiting high-risk conditions predisposing them to infection or severe manifestations of COVID-19; the vaccination protocol stipulates that inoculation during pregnancy should be scheduled after the completion of the 12th week of gestation, hence commencing anytime from the onset of the 13th week of pregnancy (65), as well as in Switzerland (66). Austria and Norway recommend COVID-19 vaccination between the 2nd and 3rd trimesters (54, 67), while Germany during the 2nd (68). Bulgaria, Estonia, and Croatia recommend COVID-19 vaccination generally for all pregnant women (69–71).

A summary of the main vaccinations offered during pregnancy in Europe is provided in Table 2.

The heterogeneous landscape of vaccination policies across European nations underscores the complex interplay between epidemiological variables, healthcare infrastructure, and regulatory paradigms. Tailored vaccination initiatives, informed by WHO directives, are progressively being enacted to address the unique requirements of the pregnant women cohort. Ranging from trimester-specific recommendations to individualized strategies in response to epidemic circumstances, national protocols underscore the necessity for adaptive vaccination approaches. Considering the heterogeneity observed in pregnancy vaccination initiatives across European nations, it becomes imperative to delineate a cohesive framework aimed at ensuring optimal maternal and fetal health outcomes via evidence-informed and collaborative policy formulations.

### 3.2 Evidence on interventions aimed at promoting pregnant women’s knowledge, attitudes, and behaviors in respect to vaccination

The initial search across MEDLINE/PubMed and Web of Science resulted in the identification of 3,186 studies. Following the removal of 1,470 duplicates and the exclusion of 1,406 studies based on the screening of titles and abstracts, a thorough full-text evaluation of the remaining 310 studies was conducted to assess their eligibility. Ultimately seven studies were included in the review, comprising three conducted in Italy (72–74), one in the Netherlands (75), one in Poland and Ukraine (76), one in Greece (77) and one in the UK (78). They encompassed a variety of research designs, including five before-after cross-sectional (72–74, 76, 77), one prospective (78), and one experimental (75) study. Four studies were conducted within hospital settings (72, 73, 76, 77). In particular, in the Italian studies, the Department of Obstetrics and Gynecology (72) and the Department of Women’s and Children’s Health and Public Health (73) organized and conducted antenatal courses; in Poland and Ukraine (76), as well as in Greece (77), the Perinatal Center and the Outpatient Clinic of the hospital carried out the perinatal visits. On the other hand, researchers in the Netherlands and in UK used online platforms for their studies (75, 78). Another Italian study adopted a hybrid approach combining hospital and online modalities due to the COVID-19 pandemic.

The recruited population across the studies comprised pregnant women participating in antenatal classes, those engaged in prenatal diagnostic consultations for congenital anomalies (72), or those attending routine prenatal visits (76, 77). The participants in the two studies conducted online were, in one case, pregnant women who signed up to the Qualtrics online panel to express interest in taking part in research activities (78), and, in the other case, pregnant women recruited through advertisement on social media (75). Sample sizes ranged from 119 (73) to 2,012 women (75), and included women between 18 and 40 years old (Table 3).

**Table 3:** Study characteristics.

Author, year	Study setting and period	Study design	Population	Sample size	Objective	Intervention	Intervention setting	Intervention tool	Tool used to assess the impact of intervention	Main results	Additional results
Januszek et al., 2022 (76)	Poland and Ukraine-Hospital-from June to August 2021	Before-after cross-sectional study	Pregnant women who attended routine pregnancy visits	300 pregnant women, including 150 Polish and 150 Ukrainian	To describe the level of vaccination acceptance, to find the factors that most influence the decision to vaccinate, and to describe the scale of changes in vaccination acceptance influenced by medical information on the safety, efficacy, and benefits of COVID-19 vaccination among pregnant women.	Physicians updated patients on current COVID-19 vaccination recommendations, safety, efficacy, and health benefits during the visit.	Medical consultations by 11 gynecologists during routine pregnancy visits were carried out at the Provincial Clinical Hospital No. 1 in Rzeszów and at the Khmelnytsky Perinatal Perinatal Center.	NA	A questionnaire, marked with a number, was administered before and after the intervention. The pre-intervention questionnaire included 30 questions around demographic details, childbirth history and miscarriages, as well as aspects related to vaccination such as safety, efficacy, side-effects severity, and frequency, vaccination status, future vaccination intentions and reasons for vaccine refusal. The post-intervention questionnaire included 18 questions that were consistent with those in the pre-intervention questionnaire, excluding the data that remained unchanged, such as age, number of deliveries, and miscarriages. Descriptive and inferential statistics were used to analyze the results.	Before physician consultations 16.7 and 35.3% of Ukraine and Poland women expressed an intention to undergo vaccination. Subsequent to gynecological consultations, there was a significant increase in the proportion of patients inclined toward vaccination, with figures rising to 46 and 72.6%. Following consultation with a gynecologist, patients exhibited significantly increased awareness of the severity of COVID-19 in pregnancy, perceived their post-vaccination immunity as better than that following infection, recognized the safety of COVID-19 vaccination during pregnancy, and expressed greater confidence in its safety. Consequently, fewer patients reported fear about receiving the COVID-19 vaccine during pregnancy.	The main factors influencing the acceptance of vaccinations were the fear of harming the fetus (OR 0.119, CI 0.039–0.324 *p* < 0.001), complications in pregnancy (OR0.073 CI 0.023–0.197 p < 0.001), and poor vaccination opportunities due to limitations in the vaccination program (OR0.026 CI0.001–0.207 p < 0.001)
Maltezou et al., 2019 (77)	Greece-Hospital-from October to December 2017	Before-after Cross-sectional study	Pregnant women who attended the Outpatient Clinic	304 pregnant women	To evaluate the knowledge about influenza and influenza vaccine and the adherence to recommendations for influenza vaccination of pregnant women	A leaflet with information about the complications of influenza was distributed to pregnant women Pregnant women also discussed with their obstetrician their concerns about vaccination.	Waiting room of the outpatient clinic at Alexandra General Hospital.	A leaflet with information about the complications of influenza during pregnancy and infancy and the efficacy and safety of influenza vaccine was distributed to pregnant women	Before the intervention, a standardized form was used to collect information about age, area of residence, immigrant, education level, number of household members, number of children <5 years old, underlying disease, number of parities, gestational age, pregnancy complications, scheduled cesarean section, smoking, intention to breastfeed, history of influenza vaccination in the past, awareness of recommendations for influenza vaccination. After the intervention a questionnaire with 11 questions was used to assess participants’ knowledge about the impact of influenza on pregnant women, neonates, and young infants and the safety of the influenza vaccine was administered. Descriptive and inferential statistics were used to analyze the results. The rate of knowledge regarding influenza and influenza vaccine was computed as [(number of correct answers)/11]*100.	39.5% of women reported that they were already informed about the recommendations to get vaccinated against influenza. Their obstetrician was the prevalent source of information (58%), followed by internet/newspaper/TV (25.5%), other healthcare professionals (25%), and friends or relatives (9.5%). 57% of pregnant women stated that they intended to get vaccinated and received a prescription; 31% of those pregnant women were not vaccinated and their main reason for not being vaccinated was “being sick” (81%)	Fear of adverse events was a frequently reported reason (27%) among women refusing vaccination followed by the perception of uselessness of vaccination (18.5%) and of being at low risk of influenza (13%). Overall, 19.5% of participating pregnant women were vaccinated against influenza at a mean gestational age of 24.6 weeks (range: 12–37 weeks, SD: 7.5 weeks)
Buursma et al., 2023 (75)	Netherlands-Online-from April to June 2020	Experimental study	Pregnant Women within 20^th^ week, speaking Dutch language, who are hesitant about accepting MPV and experience negative affect concerning the decision	382 pregnant women (151 cognitive reappraisal,107 acceptance, 124 control)	To assess whether cognitive reappraisal and acceptance are effective emotion regulation strategies to decrease the influence of negative affect on intention to accept maternal pertussis vaccination (MPV) among pregnant women	After an online baseline questionnaire (t0), two intervention groups and a control group were established. Women in the first intervention group – the cognitive reappraisal group - had to describe how they experienced the decision about MPV by trying to focus on the positive aspects of MPV decision itself. In the second intervention group - the acceptance group - women had to describe how they experienced the decision about MPV by focusing on their emotions and figuring out which emotions were triggered and why. Participants in the control group received general instructions to think about MPV decision without any specific emotion regulation instructions;	Online context	Online instructions for Cognitive reappraisal, Acceptance and Control group in English and Dutch	After the intervention participants completed a 1st post-test survey (t1); seven days later, participants were invited via e-mail to respond to the 2nd follow-up survey (t2). At all three time points (t0, t1, t2), measurements included negative affect toward the decision about MPV, attitude toward MPV, and intention to accept MPV. The impact of interventions on negative affect over time was assessed using multilevel regression	All three groups showed a significant decrease in negative affect between baseline and the follow-up, but no significant differences were found between the cognitive reappraisal, acceptance, and the control groups in changing negative affect from baseline to the first and second follow-up	NA
Costantino et al., 2021 (74)	Italy- Hospital From October 2019 to March 2020, online platform from March 2020 to October 2020	Before–after Cross sectional study	Pregnant women attending childbirth preparation courses	326 pregnant women	To evaluate the efficacy of an educational intervention to improve vaccination adherence during pregnancy	Participants took part in an educational intervention focused on maternal immunization during pregnancy, life course immunization, and vaccination recommended on the Italian Immunization Plan, conducted by healthcare professionals. At the end of the educational intervention, which usually lasted one hour, participants had the opportunity to express any doubts or concerns about the topics covered, and further vaccination counseling “on demand” was provided if requested.	Childbirth class at University of Palermo	A copy of the Vaccination Schedule of the Sicilian Region prepared by the Scientific Board of “VaccinarsinSicilia” was offered to all participants.	At baseline, participants filled in a 36 items-questionnaire, divided into five sections (demographic information and educational level; pregnancy history; self-knowledge about immunity status to Measles, Rubella, and HBV; knowledge and attitudes about influenza and DTPa vaccination during pregnancy and vaccination on early childhood). 30 days after interventions, adherence to influenza and DTPa vaccination of pregnant women was evaluated through contact by text and/or WhatsApp messages or by email address. Descriptive and inferential statistics were used to analyze the results.	After the intervention, among the responding pregnant women 47.8% received influenza vaccination (+44.8% compared to the period before the childbirth preparation course), 57.7% DTPa vaccination (+50.7% compared to the period before the childbirth preparation course) and 64.2% at least one of the two vaccinations recommended (+54.8% compared to the period before the childbirth preparation course)	A significant association was found between pregnant women who received at least one vaccination and higher educational level (graduation degree/master’s degree), employment status (employed part/full-time), and influenza vaccination adherence during past seasons (at least one during last five years)
Bruno et al., 2021 (73)	Italy- Fondazione Policlinico Universitario Agostino Gemelli IRCCS (FPG)-From October 2019 to January 2020	Before–after Cross sectional study	Women from the 4th month of pregnancy attending childbirth preparation courses	119 pregrnant women	To increase awareness and attitudes to vaccination in pregnant women, to evaluate the effectiveness of the on-site influenza vaccination offer to pregnant women (and their partners).	a 30–40 min vaccination session was held addressing the definition and mechanism of vaccines, vaccine components and classifications, adverse reactions, prevalent misconceptions, vaccination schedules during pregnancy, and access to vaccination services through the Italian National Health System, the vaccination calendar, and the mandatory vaccines in Italy.	The antenatal classes at hospital FPG	NA	Before and following the training session, participants completed a voluntary anonymous questionnaire assessing their knowledge, awareness, of vaccination, and their compliance through flu vaccination. Descriptive and inferential statistics were used to analyze the results.	Significant differences were noted in participants’ knowledge regarding the severity of infectious diseases before and after the intervention. Awareness of the severity of Hib increased from 35.63 to 54.05%, knowledge of poliomyelitis rose from 68.82 to 88.46%, and understanding of diphtheria improved from 40.45 to 61.84%. A significant change was observed in the preferences for tetanus vaccinations between the pre-and post-intervention questionnaires. During the study, 40.34% of participants received the influenza vaccination	The number of participants believing that there is no relationship between vaccination and autism rose from 41.05% in the pre-intervention to 72.97% in the post-intervention
Bechini et al., 2019 (72)	Italy- Obstetrics and Gynecology Department-From October 2017 to May 2018	Before–after Cross sectional study	Pregnant women attending childbirth preparation courses a/o prenatal diagnostic counseling on congenital defects	210 pregnant women	To evaluate pregnant women’s knowledge of and attitudes toward vaccination, their sources of vaccine information, and the impact of an educational intervention carried out by experts on vaccination	A 30-min intervention session focusing on vaccine prevention, conducted by vaccination experts Topic intervention: definition and mechanism of vaccines, concept of herd immunity, contraindications and associated risks of vaccination, detailed explanation of the National Vaccine Plan Prevention, efficacy of vaccines, recent epidemic trends, debunking of false myths, considerations regarding vaccination during pregnancy, legal aspects of compulsory vaccinations, and guidance on accessing reliable information sources.	Childbirth preparation courses or prenatal diagnostic counseling on congenital defects at the Obstetrics and Gynecology Department at the University of Florence	The intervention was supported by a set of slides, the paper version of which was then distributed to each participant	A pre-intervention questionnaire comprising sections on knowledge and attitudes toward vaccinations and the Italian vaccination program, alongside personal information including age, country of origin, and qualification was administered and followed by a post-intervention questionnaire identical to the pre-intervention. Descriptive and inferential statistics were used to analyze the results	After the intervention, there was a significant decrease from 43 to 13% in responses signifying a low level of knowledge about vaccines. A significant increase in knowledge of vaccines such as diphtheria, tetanus, pertussis, poliomyelitis, Hib was found between pre and post intervention. The average pre-intervention score for items related to women’s intentions regarding vaccination during pregnancy and vaccinating their children was 35.46 (95% CI 33.62–37.30), which increased to 42.57 (95% CI 41.31–43.82) post-intervention	The primary source of information regarding vaccines and vaccinations was reported to be word of mouth, followed by family doctors and mass media
Parson et al., 2022 (78)	UK-Online-from October to November 2019-form March to April 2020	Prospective before-after study	Pregnant women living in England, and not having received the flu vaccination during that flu season	411 pregnant women	To evaluate if the intervention effectively increased pregnant women’s intention to undergo influenza vaccination during pregnancy and influenza vaccine adherence	A 4-min animation was used to inform pregnant women about the risks of flu to themselves and their unborn babies, the effectiveness of the flu vaccination and its ease of administration.	Qualtrics survey software-online	4-min animation provided simple visual demonstrations of the processes involved in the pathogen infecting pregnant women, and how the flu vaccination works to disrupt it. Descriptions of the vaccine component, and how it works to protect pregnant women and unborn babies were also provided, to rectify any misconceptions, and reassure pregnant women about the safety and effectiveness of the vaccination	Before receiving the intervention and immediately afterward participants completed a short anonymous survey measuring illness risk appraisals. Six months later, a further short survey was administered to measure vaccination behavior and attitudes. Descriptive and inferential statistics were used to analyze the results	67 participants completed the follow-up survey at six months of follow-up. Of those no longer pregnant (43), 53.5% reported receiving the vaccination, while 46.5% had not. Among the 24 participants still pregnant, 62.5% had received the vaccination, while 37.5% had not, with 33.3% expressing no intention (44.4%) being uncertain, and (22.2%) intending to receive it. Additionally, of those with a higher intention to receive the vaccination 57.1% proceeded to receive it.	Participants’ perceptions of the likelihood and severity of flu during pregnancy significantly increased after viewing the animation

#### 3.2.1 Methodological quality assessment (risk of bias)

One of the included quasi-experimental studies reported a score of 5 out of 12 (64), three a score of 6 out of 12 (59, 60, 65), and two a score of 7 out of 12 (61, 63), showing all fair quality. The only experimental study included in the review (62) reported a score of 7 out of 14 being of fair quality too.

#### 3.2.2 Intervention characteristics

The educational interventions carried out exhibited heterogeneity across the studies. In five studies (72–74, 76, 77), interventions involved participant engagement with healthcare professionals. Among these, three (72–74) were conducted during antenatal classes held at varying frequencies, featuring educational sessions about vaccination and vaccines lasting 30–60 min and facilitated by highly qualified healthcare practitioners, with expertise in vaccinology. Since April 2020, one of these antenatal classes has been delivered online through digital platforms due to the COVID-19 pandemic (74).

Two interventions (76, 77) were integrated during routine prenatal visits. In the study conducted in Poland and Ukraine (76), participants were briefed on the safety, efficacy, and health benefits associated with COVID-19 vaccination by gynecologists, In the study conducted in Greece (77) participants were provided with an informational leaflet on influenza and influenza vaccination while in the waiting room of the clinic (77), followed by consultations with midwives (77).

In the study carried out in the Netherlands (75) pregnant women were randomly assigned to one of the 3 online groups (cognitive reappraisal intervention group, acceptance intervention group, and control group) to evaluate the influence of negative affect on intention to accept maternal pertussis vaccination (MPV). The cognitive reappraisal group was instructed to describe their experience relating to the decision regarding MPV, with specific attention to its positive aspects. The acceptance group received instructions to describe their emotional experience related to the MPV decision, trying to identify the emotions triggered and their causes. Finally, the control group received general instructions to reflect on the decision regarding MPV, without a specific focus on emotion regulation.

In another study (78), carried out online, the intervention comprised a 4-min animated video designed to inform pregnant women about the risks posed by influenza to both themselves and their unborn babies, as well as to elucidate the efficacy of the flu vaccine and its ease of administration.

#### 3.2.3 Tools for assessing the impact of intervention

In all the studies, questionnaires were used to evaluate the impact of the interventions. One Italian study (73) used a pre-and post-intervention questionnaire adapted from a validated tool (79) to assess knowledge, awareness of vaccination, and compliance to influenza vaccination. In another Italian study (72), a pre-and post-intervention non-validated questionnaire was employed, encompassing demographic details (age, country of origin, and educational attainment) alongside inquiries about participants’ knowledge and attitudes toward vaccinations, as well as their awareness of the Italian vaccination schedule. The pre-post intervention questionnaires in both studies (72, 73) included questions about participants’ knowledge and attitudes toward vaccinations; however, the specific focus and detail of these questions differed between studies. In the third Italian study (74), the pre-intervention survey was performed through a questionnaire validated in a preliminary pilot study, while the post-intervention assessment was performed by text message and/or WhatsApp message or e-mail contact and was aimed to evaluate adherence to flu vaccination and/or diphtheria–tetanus–pertussis acellularis (DTPa), as well as the main reasons for refusing vaccination.

Also, the studies conducted in Poland and Ukraine (76) and the UK (78) adopted a pre-post-intervention non-validated questionnaire survey, measuring safety, efficacy, side-effects severity, and frequency of vaccinations (76) and illness risk appraisal (78) respectively; both studies explored vaccination attitudes, one conducting the assessment immediately following the educational intervention (76) and the other six months after the intervention (78). In the investigation undertaken in Greece (77), a standardized non-validated questionnaire with 11 questions was employed to assess pregnant women’s understanding of influenza and their compliance with influenza vaccination after the educational intervention. The study undertaken in the Netherlands employed a survey administered at baseline, alongside two subsequent post-intervention surveys, to assess the impact of negative affect on the intention to accept MPV (75).

#### 3.2.4 Results

##### 3.2.4.1 Effects on knowledge

Pregnant women’s knowledge about vaccines and vaccine-preventable diseases was assessed in six (72–74, 76–78) of the included studies.

The evidence showed that the main sources of vaccination information were obstetricians (58%) (77), independent research (52.9%) (73), word of mouth (friends, family members, etc.) (9.5–50%) (72, 77), traditional mass media (TV, radio, and newspapers, internet) (19.5–35.7%) (72–74, 77), health professionals, particularly family doctors (25–45.7%) (72, 74, 77). Specialists such as pediatricians and gynecologists were consulted less frequently (16.2–21.4%) (72). Additionally, within a study carried out in Italy (73), post-intervention questionnaires revealed that 64.6% of respondents (51/79) deemed the prenatal course highly beneficial for information acquisition, showing a significant increase compared to the pre-intervention questionnaire results (30.3%, 27/89 respondents).

The level of knowledge regarding the recommendation for influenza vaccination during pregnancy exhibits considerable variability among pregnant women. In a study conducted in Italy (74), in the pre-intervention, approximately 70% of the interviewees were aware of the recommendation for influenza vaccination during pregnancy, but only 23.9% demonstrated awareness that influenza vaccination during pregnancy could be administered throughout all trimesters of gestation. Furthermore, 58.6% were aware of the recommendation of DTPa vaccination during pregnancy, but 54.6% did not know the correct timing for vaccination during pregnancy, while only 32.8% knew about the necessity of receiving a DTPa vaccine booster in each pregnancy. In a study conducted in Greece (77), in the post-intervention, 39.5% of the participants reported being already informed about the recommendations for influenza vaccination. The same study found that the average knowledge score on influenza and influenza vaccination, after the intervention, was 87% (77). However, neither the Italian nor the Greek studies evaluated the impact of the intervention on knowledge through a pre-post comparison (74, 77).

Furthermore, regarding information on vaccine-preventable diseases, in the study carried out in Poland and Ukraine (76), only 28.1% of the participants in the pre-intervention declared having received information regarding COVID-19 vaccination from their healthcare provider.

The evidence shows a low level of general knowledge about vaccinations against infectious diseases in the pre-intervention, as demonstrated by 43% of responses indicating poor or insufficient level of knowledge (72); following the educational intervention there was a notable 30% decrease in responses indicating a low level of knowledge in the vaccination field (72).

In terms of understanding the risks associated with infectious diseases, the findings indicate that, before the educational intervention, only 36.5% of participants were aware of the possible complications resulting from pertussis in newborns, and as many as 42.9% were uninformed about the potential repercussions of severe complications of influenza on both the mother and the fetus, as well as the newborn (74).

Moreover, it was revealed that 35.63% of respondents in the pre-intervention questionnaire, perceived influenza as quite serious, while almost 54% of the women in the post-intervention questionnaires shared this perception (73), with a notable increase. A significant increase in participants’ perception of the severity of influenza during pregnancy was also found following the educational intervention conducted in the British study (78).

The data showed that before the intervention, a notable proportion of women (40.5%) regarded diphtheria infection as very severe (73). Following the intervention, there was a significant increase in the proportion of women (61.8%) who perceived the infections as highly severe (73). Furthermore, after medical consultation, participants exhibited significantly heightened awareness regarding the severe clinical manifestations of COVID-19 infection (76).

Regarding vaccine safety, during the pre-intervention of one of the Italian studies (72), 15% of participants reported direct or indirect personal experiences with one or more post-vaccination adverse effects, including severe conditions such as autism, meningitis, deafness, polio, and acute leukemia. However, following the intervention, there was a reduction in this percentage, suggesting that the instances reported in the pre-intervention survey were possibly influenced by unsubstantiated beliefs or misinformation rather than genuine personal experiences.

Two studies conducted in Italy (72, 73) revealed a significant rise in the percentage of individuals who disregarded the existence of a causal association between vaccines and autism after the intervention, escalating from 43.8% (72) and 41% (73) during the pre-intervention to 84% (72) and 73% (73) during the post-intervention.

After the educational intervention, there was a significative increase in the proportion of individuals expressing a lack of concern regarding the adverse effects associated with vaccination (pre-intervention 33.3%, post-intervention 57.2%), believing that vaccines have mild side effects (pre-intervention 77.5%, post-intervention 97.40%) (73), and holding the belief that administering multiple vaccines simultaneously does not pose harm to the health of their offspring (pre-intervention 15.2%, post-intervention 70.1%) (72).

Noteworthy is the significant increase also in general knowledge regarding recommended pediatric vaccines, including diphtheria, tetanus, pertussis, poliomyelitis, and HIb, following the intervention (72).

In conclusion, these studies revealed a significant impact of educational interventions on pregnant women’s knowledge about vaccines and vaccine-preventable diseases. These interventions led to increased awareness of vaccination recommendations, decreased misinformation, and improved understanding of the severity of vaccine-preventable diseases.

##### 3.2.4.2 Effects on attitudes

Six (72, 73, 75–78) out of the seven studies included in the analysis provided insights into the attitudes of pregnant women toward vaccination for themselves.

In an Italian study (72), the mean score quantifying the inclination to vaccinate during pregnancy was 35.46 (95% CI: 33.6–37.3) before the intervention and 42.57 (95% CI: 41.3–43.8) after the intervention. Considering that the score was calculated assigning a value of “0” to responses indicating opposition to vaccination, a value of “1” to neutral or hesitant responses, and a value of “3” to responses showing a support to vaccination, the results showed a shift toward a greater support to vaccination (72).

In another study conducted in Italy, an examination of the expressed preferences for vaccinations against individual infectious diseases revealed a significant surge in the inclination toward tetanus vaccination, with an increase from 80.77 to 91.14% (73).

Following the educational intervention, a notable increase was discerned in the responses concerning women’s intentions to undergo several vaccinations for themselves, including diphtheria and pertussis (72, 73).

A significant increase in the inclination to undergo influenza vaccination during pregnancy was highlighted in the study conducted in the UK (78) at the first follow-up assessment after the educational intervention. Moreover, within this study, both the probability of contracting influenza during pregnancy and the intention to receive the influenza vaccine emerged as significant positive predictors of influenza vaccination (78). Among the cohort of 411 participants in this study (78), 67 individuals completed the second follow-up. Within this subset, 57.1% of the participants who exhibited an increased intention to undergo vaccination (with a score of ≥6 out of 10) during the initial follow-up, subsequently received the vaccine (78).

In the investigation conducted in Greece (77), 57% of the participants expressed the intent to receive the vaccine and were accordingly prescribed it. However, despite the expressed intention and prescription, a substantial portion, comprising 31% of the individuals, did not proceed with vaccination. The predominant reason cited for non-adherence was “being sick,” as reported by 81% of women who had not been vaccinated.

A significant escalation in the intention to receive vaccination is evidenced also in the study conducted in Poland and Ukraine (76). Before medical consultations, 35.3% of patients in Poland and 16.7% of patients in Ukraine indicated their plans to undergo COVID-19 vaccination. Following medical consultations, the percentage of patients expressing willingness to receive vaccination surged to 72.6% in Poland and 46% in Ukraine. The data also showed that participants with higher education exhibited significantly greater level of vaccination acceptance compared to women with lower one (76). The investigation additionally underscored that heightened resistance to vaccination and incidence of patient-perceived post-vaccination complications corresponded with the diminished likelihood of altering the decision regarding COVID-19 vaccination after medical consultation (76). Predictors of reduced likelihood of vaccination included apprehension regarding fetal harm, perceived post-vaccination complications, and limitations in vaccinations program offered (76).

The study carried out in the Netherlands (75) demonstrated that an elevated magnitude of negative affects is markedly linked to a diminished inclination to embrace pertussis vaccination. Furthermore, within this study, all 3 groups, cognitive reappraisal intervention group, acceptance intervention group and control group, exhibited a noteworthy decrease in negative affect, with no notable disparities observed among them (75). Furthermore examining the written responses provided by participants across all groups, the adoption of emotional acceptance emerges as a promising approach in alleviating the influence of negative affect on the intention to accept pertussis vaccination (75).

In conclusion, the studies results revealed a notable shift toward greater acceptance and intention to vaccinate among pregnant women, influenced by educational interventions, medical consultations and emotional regulation strategies.

##### 3.2.4.3 Effects on behavior

Following the educational intervention, a notable increase in adherence to influenza vaccination was observed across four studies (73, 74, 77, 78).

**Figure 1:**
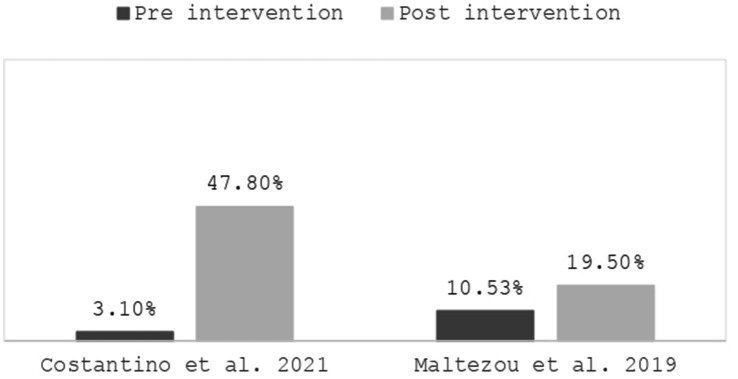
Influenza vaccine adherence.

In two studies, conducted, respectively, in Italy (74) and Greece (77), 47.8% of respondents in the follow-up (74) and 19.5% of participants (77) reported having been vaccinated post-intervention, compared to 3.1% (74) and 10.53% (77) in the pre-intervention, indicating a significant increase (Figure 1).

In two studies conducted in Italy (73) and the UK (78), respectively, 40.34% of participants (73) and 57% of respondents (78) reported receiving influenza vaccination after the educational intervention.

The empirical findings suggest that after the implementation of the educational intervention, a significant augmentation in adherence to DTPa vaccination was observed, with rates escalating from 7.4 to 57.7% (74).

Factors influencing vaccination behavior were also addressed in the included studies. In two of them (74, 77) a significant association was also found between adherence to recommended vaccinations and a higher level of education. Indeed, findings from a study conducted in Italy emphasized that individuals with a higher level of education (bachelor’s/master’s degree) exhibited notably greater adherence to recommended vaccinations in comparison to counterparts with lower educational attainment (high school/primary-secondary school diploma) (adjusted OR = 3.12; 95% CI 1.25–4.67) (74). The aforementioned findings are corroborated by those from the investigation undertaken in Greece, wherein a demonstrably significant correlation was established between higher educational attainment (college-university level) and heightened compliance with vaccination protocols (77).

Evidence also indicated that a thorough understanding of influenza and influenza vaccine, and prior influenza vaccination history, were significantly associated with an increased likelihood of receiving influenza vaccination during pregnancy [respectively OR from 1.69 (74) to 17.8 (77), and from 3.6 (77) to 4.12 (74)], in contrast to individuals lacking adequate knowledge regarding influenza and the flu vaccine, as well as those who have not received vaccinations in preceding years.

Despite the implementation of educational interventions, various factors contributed to women’s reluctance to undergo vaccination during pregnancy, as evidenced by findings from three studies (74, 76, 77).

In the study conducted in Poland and Ukraine (76), participants cited concern about fetal harms and post-vaccination complications/adverse reactions, with fear being a key emotional driver influencing their decision to avoid the COVID-19 vaccine. These concerns decreased significantly after the intervention.

Additionally, in two separate studies (74, 77), post-intervention data revealed that 47.6% (74) and 27% (77) of participants who cited reasons for refusing influenza vaccination identified fear of adverse events as the main deterrent. In a study conducted in Italy (74) the secondary predominant reason for vaccine refusal was the absence of recommendations from gynecologists/obstetricians, highlighting the pivotal role of healthcare professionals in addressing vaccination hesitancy. Additionally, the belief that influenza vaccination is unnecessary and that the risk of contracting the flu is low has been cited as additional reason for vaccine refusal (77).

In conclusion, the educational intervention led to a significant increase in vaccination adherence across several studies. Higher education levels were associated with greater adherence to recommended vaccination regimens. However, despite these positive outcomes, vaccine hesitancy persists among pregnant women, emphasizing the continued need for interventions and the crucial role of healthcare professionals in addressing concerns.

## 4 Discussion

The primary objective of this investigation was to provide an examination of the latest national vaccination policies for pregnant women in European countries and to ascertain the effects of educational interventions targeted at pregnant women on their knowledge, attitudes, and behaviors regarding vaccination within the European setting.

In each country, vaccination policies may be shaped by disparities in the incidence of vaccine-preventable diseases, vaccination adherence rates, costs, and criteria used to issue recommendations and assess potential reimbursement (80, 81). Vaccine characteristics, such as efficacy or effectiveness and safety, are critical in shaping vaccination policies, as they directly influence public health outcomes and disease prevention strategies (81). Equally important is vaccine acceptability, which affects public uptake and the success of vaccination programs. If a vaccine is not widely accepted, its impact may be limited despite its efficacy (81). Additionally, vaccination policies must consider alternative interventions, such as public health campaigns or treatments, to ensure a balanced approach to disease prevention (81). The complex interaction between these factors could be reflected in the diversity in vaccination policies between European countries (18, 37, 62–65, 82–84). Despite this, following WHO guidelines (13–15), tailored vaccination programs are increasingly being implemented. From trimester-specific recommendations to personalized strategies during epidemics, national protocols highlight flexible vaccination approaches in pregnancy.

However, given the European decreasing confidence in vaccines (85), it would be useful to establish cohesive and harmonized pregnancy vaccination strategies across European countries to promote optimal outcomes in terms of maternal and fetal health. A viable approach to harmonize vaccination recommendations across Europe, while accounting for national variations, would involve the establishment of a transparent and common, yet adaptable, European framework to identify a core set of priority recommended vaccines while allowing individual countries to integrate additional vaccines according to their specific epidemiological circumstances. In this light, ongoing and systematic monitoring would facilitate timely adjustments to the core set of recommended vaccines, ensuring it remains responsive to evolving epidemiological conditions, also in relation to specific cases. Furthermore, ensuring that information regarding vaccination schedules and local updates is readily accessible and understandable to both healthcare professionals and the public is crucial to guarantee the equity and continuity of vaccination offer, particularly for individuals traveling between countries. Transparency and standardization in decision-making processes, coupled with a thorough and regular assessment of vaccination policies are imperative to allow harmonization.

In this context, governments assume a central role in structuring and implementing evidence-based vaccination policies and strategies tailored to pregnant women and capable of responding to any specific epidemiological situation, such as a potential high circulation of the pathogen, but also to integrate with existing vaccination recommendations in the general population.

In order to enhance vaccine uptake it is of utmost importance to also address knowledge and attitudes as foundations of individual behaviors. Our review encompassed seven studies addressing these aspects through educational interventions in pregnant women. Comparability across studies was restricted owing to variations in the contexts and nature of interventions implemented, as well as the criteria and methodologies used for evaluating results. Furthermore, the generalizability of the results can be influenced by the specific context of each country. For example, countries such as the United Kingdom, Greece, Poland, and Ukraine have similar vaccination policies for pregnant women, including recommending pertussis and influenza vaccines, as highlighted in our research (18, 37). In these countries, educational interventions have been implemented (76–78) specifically to raise awareness of influenza and pertussis vaccination. Therefore, given the existing vaccination awareness promoted by national policies, one might hypothesize that an educational intervention developed in one of these countries could have similar effectiveness when implemented in another. However, substantial heterogeneity in vaccination policies across countries, coupled with variations in national health cultures and health systems, complicates the prediction of the effectiveness of educational interventions developed within one national context when applied in another. This highlights the need for a more nuanced assessment of the adaptability and effectiveness of such interventions in accordance with the unique conditions of each country. Nevertheless, we contend that a favorable inference can be derived from the findings of the studies we reviewed, albeit challenges remain also in particular with respect to the reproducibility of interventions and methodology to assess their impact.

A relevant aspect that emerged from the collected evidence is concerning primary sources of information for pregnant women that mostly encompass obstetricians and healthcare practitioners (72–74, 77). In this respect, the absence of recommendations from gynecologists/obstetricians emerged as a pivotal determinant influencing vaccine refusal from one study conducted in Italy (74). In a recent Italian survey, about one-third of gynecologists expressed safety concerns about administering the influenza vaccine during the first trimester whereas Tdap vaccination is recommended in the third trimester with less safety concern (86). Furthermore, most participating gynecologists had themselves low influenza and Tdap vaccination rates, which might have affected their confidence in recommending vaccines (86, 87). Indeed, gynecologists/obstetricians are regarded as trusted healthcare professionals during pregnancy in Italy (85), therefore their advice was shown to play a crucial role in influencing decisions regarding vaccination uptake (88). This also aligns with the evidence of the fundamental role of healthcare professionals in combating vaccination hesitancy (29, 89–92). Nonetheless, albeit vaccinations should be addressed during antenatal care, it is not certain that this is done constantly and in a standardized way. The increasing prevalence of healthcare workers declining vaccination for themselves and abstaining from recommending it to their patients (93–96) may contribute to patient vaccine refusal and the observed low rates of vaccination acceptance, as also suggested in the discussion of one of the considered studies (76). A recent systematic review of the literature on vaccine hesitancy and vaccination coverage among healthcare workers in Europe has highlighted significant variability across countries and among vaccines (97). Vaccine hesitancy varies by country, with rates of 8% among all healthcare workers in Italy and up to 40% among physicians in France. Variations are also higher in respect to COVID-19 vaccines. Eventually, despite methodological differences across studies, physicians consistently exhibited lower levels of vaccine hesitancy compared to nurses, alongside higher vaccination rates for several vaccines, including COVID-19, influenza, diphtheria, tetanus, and pertussis (97). Contributing factors to vaccine hesitancy and vaccination refusal among healthcare professionals include concerns about adverse side effects, influence from individuals in personal networks who refuse vaccination, and diminished trust in vaccines, paralleling trends observed in the general public (97). It is anyhow worth noting that not all healthcare practitioners are experts in vaccinology, and their vaccine hesitancy may stem from uncertainties or even doubts regarding potential risks, public controversies, misinformation, as well as interactions with hesitant patients (97, 98). Hence, the training and implementation of tailored educational interventions on vaccination also for healthcare professionals are deemed imperative because awareness and knowledge were also found to increase healthcare professionals’ willingness to recommend vaccination (93).

Moreover, the execution of educational interventions facilitated by healthcare professionals specially trained may serve to alleviate misinformation concerning vaccines, which may stem from traditional (99) and social (100) mass media or word-of-mouth sources (101). Indeed, mass media have the potential to exert negative effects on vaccine-hesitant populations or instead, they could be used as a vital tool for disseminating vaccination culture (99, 102), despite assertions in existing literature indicating that women place greater trust in information provided by healthcare professionals compared to that disseminated through mass media or informal communication channels (89). For this reason, an effective strategy could be represented by educational intervention, carried out through social media but by healthcare professionals. Three studies (74, 75, 78), examined in the review, exemplify a commendable utilization of media for enhancing vaccination awareness among pregnant women, employing online platforms and the internet as vehicles for educational interventions and subsequent evaluation of outcomes, showing an effective approach toward addressing vaccination awareness. In one of the included studies (75), social media platforms were leveraged for participant recruitment, thus allowing the target population to be easily reached, as prospective parents demonstrate regular activity on social media and those uncertain about their decision about vaccination tend to look for information online.

Even if vaccination refusal is usually multifactorial (103), the deficiency of information regarding the safety and efficacy of vaccines commonly catalyzes vaccination refusal (104). The results of our review showed a notable deficiency in knowledge and awareness concerning the vaccination field, specifically recommended vaccines during pregnancy (72, 74, 76, 77), vaccine-preventable diseases, and their severity for both pregnant women and offspring (72–74, 76, 78) before any educational intervention, consistent with extant literature (29, 91, 105–107).

Conversely, following the implementation of educational interventions, there was a discernible increase in comprehension within these domains, leading to an escalation in the inclination to receive vaccinations during pregnancy (72), consequently resulting in a significant enhancement in adherence to recommended vaccination recommendations (73, 76, 77). Nevertheless, caution should be paid in the interpretation of these results because it is expected that pregnant women’s knowledge about recommended vaccination increases with the increase in gestation week. Unfortunately, the specific week of pregnancy during which knowledge was assessed was not explicitly stated, except indirectly in the case of two Italian studies that reported that the most of participants were in the third trimester (73, 74).

Nevertheless, in this respect a standardized and validated curriculum should be developed to lead educational interventions and make them more comparable. This curriculum should be evidence-based and encompass vaccine-preventable diseases characteristics, recommendations for vaccination in pregnancy, and vaccines efficacy, effectiveness and safety. The curriculum could be adopted by trainers in the field as well as by all healthcare professionals engaged in prenatal care, including gynecologists, obstetricians, midwives, and nurses. A particular attention should be paid to adapt the curriculum to pregnant women’s needs and capabilities. In fact, our data also showed a general lower likelihood of vaccination during pregnancy in individuals with a low degree of education (74, 76, 77), in accordance with existing literature (108, 109). Thus, it is advisable to customize educational interventions to align with the educational and socio-demographic context of the target population, given that these variables may exert influence on vaccination decisions.

The educational intervention ought to comprehensively address not only the potential adverse effects of vaccination, debunking associated misconceptions and contrasting negative affect, i.e., fear, discomfort, anticipated regret (75), and perception of complications and damage after administration (76), but also underscore the risks associated with vaccine refusal for both the pregnant woman and her offspring, which may lead to significant complications.

The multi-component approach, incorporating educational interventions and vaccination administered by trained personnel, alongside healthcare professional training and continuous education, has exhibited superior effectiveness in enhancing maternal attitudes toward recommended vaccines during pregnancy (94–96, 98). Moreover, it has proven efficacious in augmenting vaccination adherence rates among both prenatal and postnatal women (94–96, 98). Furthermore, new methodologies, including reminder and active call systems (94, 95), as well as the utilization of digital modalities such as text, video, or audio messages, and internet-based interventions (e.g., websites, mobile applications, or social media platforms), have underscored their effectiveness in a context significantly influenced by the recent COVID-19 pandemic. This context is also marked by heightened vaccine hesitancy, alongside an overall increase in the complexity of vaccination schedules, heightened expectations from caregivers, and lifestyle changes (100).

The findings of our work should be read considering some limitations. First of all, the search strategy adopted to look for both vaccination policies in European countries and the evidence on educational intervention might have failed in identifying all relevant information also considering that some recommendations could be issued in local languages thus being difficult to find and report. Another aspect to be considered is that vaccination policies could be implemented differently between and within countries. Regarding the evidence on the impact of educational interventions, it should be noted that, because all studies relied on questionnaires, whether validated or not, the potential for social desirability bias could not be ruled out. Notably, the studies included in our review did not employ tools designed to specifically measure social desirability bias. However, the use of anonymized questionnaires in these studies may have helped mitigating this bias. Additionally, in one instance (75), being a randomized experimental study, the process of randomization may have contributed to controlling for this bias. As a matter of fact, all studies included in our work were judged of fair quality and this calls for other research in the field to better disentangle the potential impact of educational interventions also considering different contexts.

## 5 Conclusion

In conclusion, there is considerable variability across European countries regarding vaccination policies during pregnancy. Tailored vaccination policies and recommendations, aligned with WHO guidelines, reflect the diverse epidemiological contexts and healthcare systems of individual countries.

Educational interventions carried out to promote pregnant vaccination by increasing knowledge and changing attitudes varied in approach and context so far. Nonetheless, they collectively demonstrated significant impacts on pregnant women’s vaccination-related knowledge, attitudes, and behaviors in Europe. From antenatal classes to online platforms and informational leaflets, these interventions led to increased awareness of vaccination recommendations, reduced misinformation, and improved understanding of the severity of vaccine-preventable diseases. Indeed, pre-intervention assessments revealed gaps in knowledge and concerns about vaccine safety, but post-intervention, there was a notable improvement, leading to enhanced adherence to recommended vaccination protocols.

Healthcare professionals emerged as the most trusted source of vaccination information, highlighting their crucial role in addressing vaccine hesitancy.

Attitudes emerged as a significant predictor of intention to vaccinate, with positive attitudes associated with stronger intentions. Emotional regulation strategies also played a role in increasing vaccination acceptance.

Behaviorally, there was a significant increase in adherence to influenza and DTPa vaccination post-intervention, particularly among those with higher education levels. However, vaccine hesitancy persisted among some, driven by concerns about adverse events and a lack of recommendations from healthcare professionals.

Overall, the findings of this investigation underscore the importance of strengthening the process behind the development of evidence-based vaccination policies and the need for specific educational interventions to increase vaccination acceptance and optimize maternal and fetal health outcomes in the European context. Further research and collaborative efforts are warranted to address barriers and facilitators to vaccination uptake among pregnant women.
